# Geographic isolation drives speciation in Nearctic aphids

**DOI:** 10.1038/s42003-022-03771-5

**Published:** 2022-08-08

**Authors:** Nate B. Hardy, Chloe Kaczvinsky, Gwendolyn Bird, Robin Richter, Jeremy R. Dettman, Eric Maw, Bryan M. T. Brunet, Robert G. Foottit

**Affiliations:** 1 Department of Entomology and Plant Pathology, Auburn University, Auburn, AL USA; 2 Department of Zoology, University of Oxford, Oxford, England UK; 3 Ottawa Research and Development Centre, Agriculture and Agri-Food Canada, Ottawa, ON Canada

**Keywords:** Evolution, Entomology

## Abstract

Across herbivorous insect clades, species richness and host-use diversity tend to positively covary. This could be because host-use divergence drives speciation, or because it raises the ecological limits on species richness. To evaluate these hypotheses, we performed phylogenetic path model analyses of the species diversity of Nearctic aphids. Here, we show that variation in the species richness of aphid clades is caused mainly by host-use divergence, whereas variation in speciation rates is caused more by divergence in non-host-related niche variables. Aphid speciation is affected by both the evolution of host and non-host-related niche components, but the former is largely caused by the latter. Thus, our analyses suggest that host-use divergence can both raise the ecological limits on species richness and drive speciation, although in the latter case, host-use divergence tends to be a step along the causal path leading from non-host-related niche evolution to speciation.

## Abstract (web summary)

Using phylogenetic path modelling, geographic isolation is revealed to be the main driver of Nearctic aphid speciation, with subsequent host-use divergence raising the limits on clade species richness.

## Introduction

One out of every three species is an herbivorous insect^1^. How did they become so diverse? Although biologists tend to think of speciation as being driven by geographic isolation between subpopulations^2,3^, the diversification of herbivorous insects is more often thought to be driven by divergent selection on host-use, stemming from co-evolutionary antagonism with their host plants^4–6^.

Two general features of the relationships between herbivorous insects and their host plants would seem to make speciation via host-use divergence especially likely. First, the relationships tend to be specific^7^. Second, the relationships are complex. As herbivorous insects feed on their hosts, they compete with other herbivores and plant parasites, and contend with host defenses and natural enemies. Moreover, in many herbivorous insect species, host plants provide sites for mating and oviposition. With so much of their biology linked to specific host associations, divergent selection on host-use could very well cause reproductive isolation. Indeed, some of the most trumpeted examples of sympatric speciation feature herbivorous insects^8–12^, much of the classic theory of co-evolution and co-divergence was inspired by patterns of herbivorous insect diversity^4,13^, and as per predictions of this theory, comparative phylogeneticists have repeatedly found positive links between the species richness and host-use diversity of herbivorous insect clades^14,15^.

**Fig. 1:**
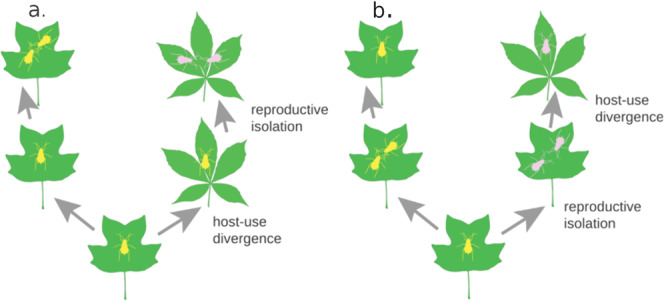
Competing hypotheses for the role of host-use evolution in herbivorous insect diversification. **a** Host-use divergence leads to the evolution of reproductive isolate, hence evolutionary antagonism is the main driver of speciation. **b** Host-divergence follows the evolution of reproductive isolation, and raises the ecological limits on species diversity.

Nevertheless, few cases of sympatric speciation via host-use divergence have been documented in detail, many predictions of the classical co-diversification theory have yet to be tested with rigor, and the positive associations between clade species richness and host-use diversity could have causes other than speciation via host-use divergence. Here, we consider one alternative causal hypothesis in particular (Fig. 1), namely, that speciation in herbivorous insects tends to be via divergence in non-host niche components linked to geographic isolation between subpopulations, and that subsequent host-use divergence raises ecological limits on species diversity^16–18^. More concretely, host-use divergence determines the extent to which herbivorous insect species can coexist in a community—that is, the overall force of competitive exclusion—as well as the overall geographic extent of an herbivorous insect clade—essentially a composite of the geographic ranges of their host plants. If this is the case, we would still expect strong positive links between species richness and host-use diversity, but there would be less correspondence between rates of speciation and rates of host-use divergence. Of course, these hypotheses are not mutually exclusive; host-use divergence could both drive speciation and raise the limits on species richness.

So, how does host-use divergence tend to affect the species richness of herbivorous insects? Does it tend to drive speciation? Or is it more important in boosting the limits on species richness? For insight, we performed a comparative analysis of Nearctic aphids for which we have exceptionally detailed views of host-use and geographic distributions. We inferred their phylogenetic relationships, characterized their niches, and used phylogenetic path models to estimate causal relationships between speciation and niche component evolution. We found that host-use divergence both drives speciation and raises the limits on species richness. But evolutionary divergence in host-use tends to be driven by divergence in non-host-related niche components, which have greater combined direct and indirect effects on speciation.

## Results

We considered two sets of models. In one set, we looked at what causes variation in the species richness of aphid genera. In a second set, we looked at what causes variation in speciation rates across the branches of the aphid phylogeny. To reiterate, disparities in species richness across clades can be caused by differences in net speciation rates, or differences in the limits on species richness. Our first set of models can identify niche components that limit species richness; our second set can identify niche components that drive speciation. A few words about the characterization of non-host niche components will help with interpretation. In broad strokes, we correlated the spatial distribution of aphid species with that of environmental variables such as soil type, elevation and primary productivity. Such variables tend to be spatially auto-correlated, so divergence along non-host niche axes will tend to entail geographic divergence^19,20^. But it is theoretically possible for non-host niche divergence to take place without obvious geographic isolation—for example, if environmental patches interdigitate—or for populations to be spatially isolated but occupy otherwise indistinguishable environments. So, our models explicitly distinguish between host-use and non-host niche components, and provide indirect evidence about the role of geographic isolation. See the “Methods” for further detail.

**Fig. 2:**
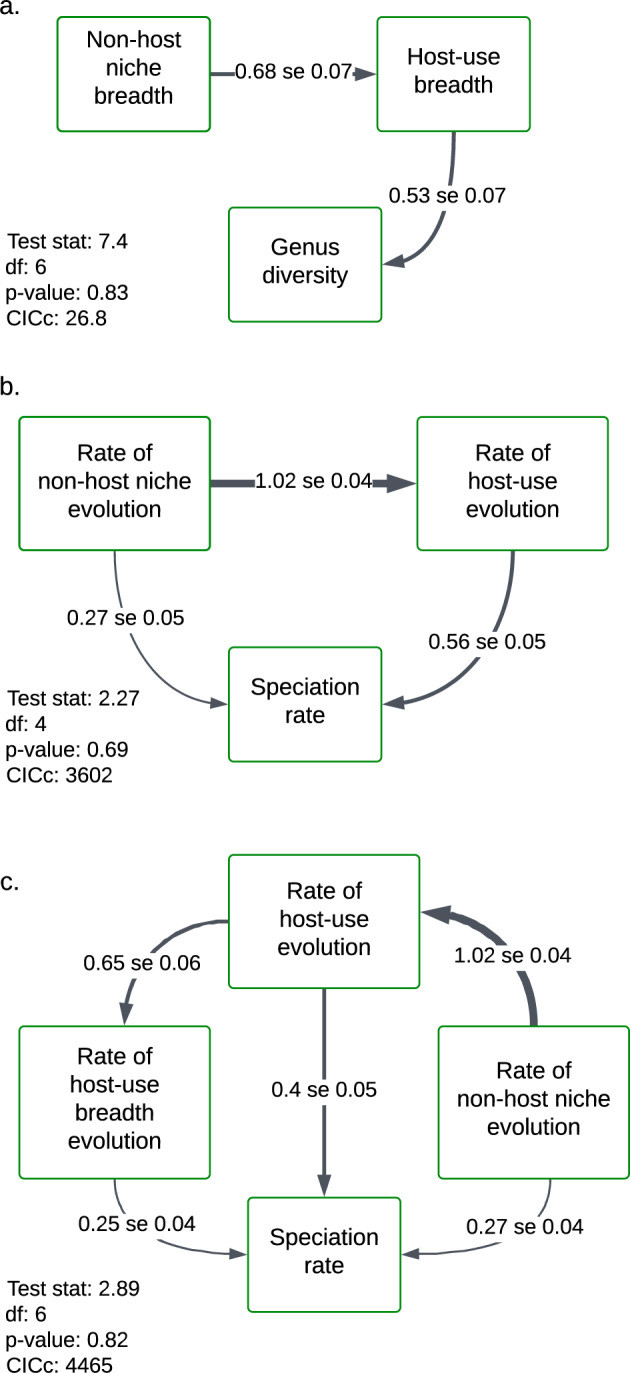
Path models showing causal relationships between the evolution of niche components and species diversity in Nearctic aphids. Values on edges are standardized mean differences for the combined effects of the first two principal components for variables representing environmental niches and host-use niches. Arrow width indicates relative strength of effect. The *p*-value provided for each model is for a Chi-squared tests of goodness-of-fit; as a rule of thumb, values >0.5 indicate a good fit to the data. **a** Best model of the causes of variation in the species richness of aphid genera. **b** Best model of how host-use evolution and environmental niche evolution affect speciation rate. **c** Best model of how the host-use evolution, host-use breadth evolution, and environmental niche evolution affect speciation rate.

Let us look first at what causes the variation in the species richness of aphid genera. We considered two alternative causal hypotheses, one in which the diversity of host-use niches and non-host-related niches could each directly affect the species richness of aphid genera, and a second in which the diversity of non-host-related niches could only affect aphid species richness indirectly, through its effects on host-use niche. We found that the latter was a better fit to the data (CICc difference = 26.8): non-host-related niche diversity positively affects host-use diversity, but does not directly affect the species richness of aphid genera. The direct effect of host-use diversity on the species richness of aphid genera was 0.53 standard mean differences (SMD) (±0.07 se), the direct effect of non-host-related niche diversity on host-use diversity was 0.68 SMD (±0.07 se), and the indirect effect of non-host-related niche diversity on aphid species richness—calculated as the product of path coefficients—was 0.36 SMD (±0.07 se) (Fig. 2a). Variation in the extant diversity of aphid clades is caused more by host-use diversity than by non-host-related niche diversity.

Let us now turn to the causes of variation in speciation rates. We first considered each special form of a general model in which (1) the rates of host-use evolution (changes in location in the host-use component space) and non-host-related niche evolution each could directly affect aphid speciation rates, (2) the rates of non-host-related niche evolution and host-use evolution were correlated—that is, each could potentially cause variation in the other, and (3) host-use evolution could potentially affect speciation rates indirectly via its effects on non-host-related niche evolution, and vice versa. In the best-fitting model (Fig. 2b; AIC difference = 4465), the direct effect of non-host-related niche evolution on speciation was 0.27 SMD (±0.05 se), while its indirect effect (through its effects on host-use evolution) was 0.57 SMD, with a total effect of 0.84 SMD. The direct effect of host-use evolution on speciation was 0.56 SMD (±0.05 se). Thus, the combined effects of non-host-related niche evolution on speciation are greater than those from host-use evolution. The proximate cause of aphid speciation is more often host-use evolution than non-host-related niche evolution, but host-use evolution tends to be caused by divergence in non-host niche components.

We then considered models in which the rate of host-use breadth evolution was included along with the rate of evolution of host-use location in the component space, following hypotheses in refs. ^21–23^. Of these models, the best-fitting (AIC difference = 3602; Fig. 2c.) had a direct effect of non-host-related niche evolution on speciation of 0.27 SMD (±0.04 se), and a total indirect effect of non-host-related niche evolution on speciation of 0.57 SMD. Hence, the total effect—taking the product of coefficients along each path, and then summing effects along each path from one variable to another—was 0.84 SMD. The direct effect of host-use evolution was 0.40 SMD (±0.05 se), with additional indirect effects through the evolution of host-use breadth 0.16 SMD, with a total effect of 0.56 SMD. The direct effect of host-use breadth evolution on speciation rate was 0.25 SMD (±0.04 se). In summary, as in models that ignore variation in host-use breadth, increasing the rate of host-use evolution increases the net speciation rate, but the rate of host-use evolution itself is determined by the rate of non-host-related niche evolution. By and large, speciation starts with non-host-related niche divergence.

## Discussion

These analyses of Nearctic aphids, although taxonomically constrained, constitute the most comprehensive tests to date of the classical hypothesis that herbivorous insect speciation is driven primarily by host-use divergence. We pitted this hypothesis against an alternative, to wit, that following speciation by other means, host-use divergence raises ecological limits on the species richness of aphid clades and communities. We found that although there is evidence for both processes, the latter appears more important: whereas the disparities in species richness across aphid genera are explained more by host-use divergence than non-host-related niche divergence, the opposite is true of the disparities in speciation rates across aphid lineages. Thus, it appears that speciation in aphids tends to start with divergence in non-host-related niche components that would be expected to diverge with geographic isolation, and host-use divergence primarily shapes aphid species diversity by raising the ecological limits on species richness. That being said, we did find evidence of speciation via host-use divergence per se. Thus, returning to our initial question—of why herbivorous insects are so species rich—our analyses suggest that it is because speciation can be caused by both geographic isolation and host-use divergence, which can also increase the conservation of species diversity resulting from either process.

This is not the first indication that the importance of speciation via host-use divergence in herbivorous insects may have been somewhat overstated. In particular, previous researchers have noted that the phylogenetic pattern of host-use in some aphid subcaldes^24–26^, and some non-aphid clades^27^, suggest that less than half of all speciation events are linked to a major shift in host use. Our analysis builds on this work, by considering more complex models of herbivorous insect niches, and performing more explicit tests of competing causal hypotheses about the ecology of herbivorous insect speciation. Although we did not directly model the phylogenetic evolution of geographic ranges, or use such models to demonstrate a direct link between geographic range evolution and speciation, we did show that speciation is driven by divergence of non-host-related components of aphid niches, such as net primary productivity and soil type, that are spatially auto-correlated and thus likely do diverge through geographic isolation^19,20^. Moreover, our models of the non-host-related components of aphid niches rest on correlations between aphid species occurrence in geographic and environmental space; hence, there is a fundamental concordance between non-host-related niche divergence and geographic isolation.

The degree to which our inferences apply to other groups of herbivorous insects may be limited by the fact that in many ways aphids are unusual^28^. For example, many aphid species have unusually complex life cycles and high reproductive rates^29,30^. Further study of other groups of herbivorous insects should give us a better sense the generality of our results. Nevertheless, what we found in aphids is consistent with what one might expect for most non-herbivorous-insect groups: speciation tends to be a geographic process, with niche divergence in isolated populations increasing the odds of species co-existence when populations converge.

## Methods

### Aphid phylogeny

Despite their economic importance as agricultural pests—in particular as vectors of plant diseases—prior to this study the aphid phylogeny was poorly resolved^28^. A few genera have been studied in detail^31,32^, but previous estimates of supra-generic relationships have been based on DNA sequence data from only a few genetic loci and species^33–35^. Such data have proven insufficient to infer deep phylogenetic relationships with any confidence. To improve on that situation, we obtained a large DNA sequence data set with Ultra-Conserved Element (UCE) target-enriched genomic sequencing of 454 aphid samples, covering 403 taxa collected across Canada and the United States. Specimen data are given in Supplementary Data 1. Samples cover each of the three aphid families (Aphididae, Adelgidae, and Phylloxeridae) along with 14 of 24 of the nominal subfamilies of Aphididae. Two psyllid species were included as outgroups for rooting.

### DNA isolation

DNA was extracted using either Omega E.Z.N.A. Tissue DNA Kits (Norcross, GA) following manufacturer guidelines or Qiagen DNeasy 96 Blood and Tissue Kits (Toronto, ON) with modifications following^36^ and elution into 2 × 20–25 μl volumes to increase DNA yield. The abdomen of each specimen was punctured by a single pin prick and the whole body used for overnight lysis of tissues. Following DNA isolation, intact specimen cuticles were retained as vouchers and deposited in the Canadian National Collection of Insects, Arachnids and Nematodes. DNA concentration was quantified using the double-stranded high sensitivity assay for the Qubit 3.0 Fluorometer (Invitrogen), and where appropriate, 10 μl of DNA of up to four specimens from a single sample was pooled.

### Library preparation, target enrichment, and sequencing

DNA libraries were prepared, enriched and sequenced at either Arbor BioSciences (Ann Arbore, Michigan) or Agriculture and Agri-Food Canada’s (AAFC) Molecular Technologies Laboratory (MTL, Ottawa, ON). At Arbor BioSciences, libraries were prepared with Illumina Nextera kits with iTru Y-yolk adapters^37^ following standard protocols, and sequencing was on the Illumina NovaSeq S4 platform. At MTL, libraries were prepared using NEBNext Ultra II FS DNA library prep kits (New England BioLabs, Ipswich, MA), also with iTru Y-yolk adapters. Library preparation steps used half reaction volumes (except where noted), but otherwise followed manufacturer guidelines with a few optimizations. DNA was sheared by enzymatic fragmentation to a length of 200–450 bp after a 15 min incubation. Adapters were diluted to either 0.6 μM (<40 ng of DNA input) or 1.5 μM in TLE as recommended for 5–99 ng DNA inputs. Adapter ligated inserts were purified via a 1X SPRI bead wash and eluted into 17 μl of 0.1X TE. For all cleanup steps, a generic SPRI bead substitute was used (Sera-Mag Select, GE Healthcare) in combination with a high-throughput rare earth magnetic stand (Invitrogen DynaMag™-96 Side Magnet). PCR enrichment with iTru dual index primers (10 μM) proceeded after tailoring index PCR thermal profiles according to the total amount of DNA input such that libraries prepared with ≤40 ng of DNA input received 9 cycles of PCR, those with 40–100 ng received 7 cycles, and those with unquantifiable DNA concentrations received 9–12 cycles. PCR products were then purified with a 1X SPRI bead wash and eluted into 17.5 μl of 0.1X TE.

Post-PCR libraries were enriched for 2731 Ultra-Conserved Elements (UCEs) using 40,207 baits (UCE Hemiptera myBaits – Hyb Capture kit, Arbor BioSciences) designed by Faircloth^38^. To decrease sequencing costs, 8–10 libraries were multiplexed at equimolar ratios for a total of 50–500 ng per enrichment pool following fluorometric quantification. Enrichment reactions followed manufacturer’s guidelines except the amount of biotinylated RNA probes was decreased to 2 μl per reaction before a 24 hr hybridization at 65 °C. Post-capture library pools were amplified with an on-bead approach using Illumina PCR primer cocktail (20 μM) and a 1 min PCR extension step. PCR products were assessed for quality via Qubit and 4200 TapeStation High Sensitivity D1000 assays, as well as qPCR (KAPA Library Quantification Kit) on a Roche LightCycler 480, and then pooled (*n* = 41–72) at equimolar ratios and loaded at 8–10 pM with 5% phiX in four runs on an Illumina MiSeq using 600 (v3) cycle kits.

### Sequence assembly, alignment, and analysis

Raw Illumina reads were deposited in the NCBI SRA repository under BioProject PRJNA819460. Reads were assembled using the Snakemake^39^ pipeline developed for analysis of target enrichment data (https://github.com/AAFC-BICoE/snakemake-partial-genome-pipeline) and described in detail elsewhere^40^. In brief, reads were trimmed using BBDuk (https://sourceforge.net/projects/bbmap/) and assembled using four approaches: Abyss^41^ with and without prior merging of paired-ends using BBMerge^42^, SPAdes^43^, and rnaSPAdes^44^. Phyluce^45^ was used to identify and filter UCE loci from the assemblies with probe sequences as input and default parameters for minimum identity and coverage. Only the longest fragment for each locus across all assemblies was used in further analyses. For each locus, we perform a multiple sequence alignment with MAFFT^46^, and trimmed ambiguous regions with trimAl^47^. We then used a Python script (Supplementary Data 2) to concatenate trimmed locus alignments, yielding a supermatrix of ~400,000 sites, spanning more than 1000 loci.

Phylogenetic relationships were estimated with maximum likelihood optimization with RAxML^48^, with each locus evolving under a separate general time-reversible (GTR) nucleotide substitution model with gamma-distributed among site rate heterogeneity. The tree search began with optimization of 100 non-parametric bootstrap data sets. Then every fifth optimal bootstrap tree was used as a starting point for optimization of the observed DNA data. With this approach, we estimated a phylogeny with strong statistic support (Supplementary Data 3; 417 of 452 nodes with non-parametric bootstrap values >90%). The implications of our estimate for aphid systematics will be dealt with in another paper. Here suffice it to say, that we found that most of the currently recognized supra-generic taxa are monophyletic.

To further extend the taxonomic breadth of our view of aphid phylogeny, we used the UCE-based tree estimate as a back-bone constraint in a FastTree 2^49^ estimate with a global GTR nucleotide substitution model and based on COI sequences of 401 species that had been obtained for DNA barcoding, including 77 species not represented in the UCE data set (Accession data given in Supplementary Data 4, and the tree in Supplementary Data 5).

### Aphid Niches

To characterize the host-use niches of Nearctic aphid species, we combined data from published catalogs^50–52^ provided in Supplementary Data 6 with data from aphid specimen collections (details given below). (Note that because aphids are sessile and colonial, our view of the associations between aphids and their host plants is relatively unbiased by incidental and ecologically trivial observations). We used these data to make a qualitative incidence matrix of the use by aphid species of host plant families. For a more efficient coding of host use, we used the R package *vegan*^53^ to calculate Jaccard distances from the host-use incidence matrix, and then subjected the distance matrix to principle coordinates analysis. We used the first two principle coordinates in subsequent analyses, accounting for 34% of the variation in host-use in our models of the species richness of aphid genera, and 40% of the variation in our models of speciation rates. These coordinates provide an address for each aphid species in a multivariate host-use space. In addition to this addressed location of a species’ host-use niche, we were also interested in the breadth of each species’ host associations. We expressed this with Shannon’s Diversity Index, calculated with the R package *vegan*. To be clear, we did not attempt to model how aphid species apportioned host tissues.

To model the non-host-related aspects of the niches of aphid species, we correlated the spatial distribution of aphid specimens with that of several environmental variables (with scripts provided as Supplementary Data 7 and 8). To 42,935 of our own aphid specimen occurrence records (Supplementary Data 9), we added 166,924 records obtained from the Global Biodiversity Information Facility (gbif.org) and iDigBio (idigbio.org); the combined occurrence data are provided in Supplementary Data 10. Irregularities in species names were resolved using the R package *rgbif*^54^. Only aphid taxa with 10 or more occurrence data were considered, a level of sampling at which predictive species distribution models tend to achieve reasonably high accuracy^55^. This left us with 372 species. We considered several variables that could affect the suitability of habitats for aphids and their host plants. Climate data were obtained from Worldclim 2^56^. From MODIS, we took data on elevation, net solar radiation, net primary production (as measured by carbon dioxide output), vegetation and leaf area indices, and land cover types. We used soil data from the Unified North American Soil Map^57^. And we took evapotranspiration data from the Consortium for Spatial Information^58,59^. We annualized environmental variables which where published as a rasters of monthly values. Using the R package *raster*^60^, for each environmental variable, we extracted values from locations at which each aphid species has been observed, at a resolution of 5 arc-min. In other words, we used the distribution of each aphid species as a mask on the maps of environmental data. For environmental variables, we calculated ranges and mean values. To avoid bias from outliers, ranges spanned the 25 and 75% quantile values rather than the minimum to maximum. We then used a principle components analysis to reduce the dimensionality of the environmental niche characterizations, using the first two principle components in path analyses, representing 34% of the variation in the species richness models (Supplementary Data 11), and 31% in the speciation rate models (Supplementary Data 12; Note the values of this tables are phylogenetically-independent contrasts, with a row for each internal nodes in the aphid phylogeny).

### Phylogenetic path models

To understand how ecological divergence affects aphid species divergence, we conducted phylogenetic confirmatory path analyses. In brief, with path models we can see if the correlations or statistical dependencies among a set of variables are consistent with those predicted by a causal hypothesis^61^, and with phylogenetic path models we can do that in a way that accounts for the non-independence of variable observations due to shared ancestry^62^. Here, we first considered the ecological causes of disparities in the species richness of aphid genera, and we then considered the ecological causes of variation in aphid speciation rates.

For the species-richness models, the species richness of each aphid genus was obtained from Aphid Species File^63^. We excluded from the richness counts any species that does not occur in the Nearctic or which has been recently introduced. We also excluded from the species richness values any species not recovered in a clade with the majority of its nominal congeners. Conversely, we did count non-congeneric species that were recovered nested within a nominal genus clade. As a rule, wherever aphid phylogeny and classification were in conflict, we gave priority to phylogeny. Likewise, when characterizing composite niches of aphid genera, we used the species included in the richness tallies. We made a version of our aphid phylogeny with just one tip branch per genus (Supplementary Data 13). We had a total of 140 genera represented. Phylogenetic path models were specified and fit with the R package *phylopath*^64^, and compared with C-statistic information criterion (CICc scores^61^. To be clear, although they are subjective constructs, we analyzed variation in the species richness of aphid genera because the generic classification is a ready-made scheme for dividing the extant aphid species richness into a set of mutually-exclusive clades.

For the speciation-rate models, we removed from the phylogeny any species for which we lacked sufficient spatial data (fewer than ten records); this left 371 species. We then calculated a branch-specific index of speciation rates—the Diversification Rate (DR) statistic of Jetz et al.^65^, using R code from Rabosky and Goldber^66^. This is the inverse of a weighted sum of branch lengths connecting a tip branch to the root of a phylogeny, with each successively more rootward branch down-weighted by a factor of 0.5. Our aim was to causally link variation in this speciation rate index to variation in the rates of niche component evolution. Thus, for each niche component, we calculated a Trait Rate (TR) statistic that is analogous to the DR statistic (see Supplementary Data 8). We reconstructed the phylogenetically ancestral states of each niche component with maximum likelihood optimization of a Brownian Motion model. Then, we summed between-node differences in ancestral trait values along the branch path from each tip to the root. This sum for each tip was the TR statistic. For the calculations, we used the R packages *phangorn*^67^ and *phytools*^68^. To control for the phylogenetic non-independence, we then calculated phylogenetic independent contrasts (PICs) for the DR statistic and each TR statistic.

Because most of our aphid niche variables exhibited considerable skew and kurtosis, prior to path modeling, variables were normalized using the R package *bestNormalize*^69^. We then specified and fit confirmatory path models using the R package *lavaan*^70^. Model fit was assessed through AIC scores and *p*-values, where a model p-value below an alpha-level of 0.05 was interpreted as indicating a missing causal relationships. In one set of models, we looked at how speciation rates were affected by rates of environmental niche evolution and host-use evolution in a multivariate host-use space. In another set of models, we considered the rate of host-use breadth evolution in addition to the rate of host-use in that multivariate space. Model scripts are provided in Supplementary Data 14 and 15. An outline of our analysis workflow is provided in Supplementary Data 16.

### Reporting summary

Further information on research design is available in the Nature Research Reporting Summary linked to this article.

## Supplementary information

Description of Additional Supplementary Files

Supplementary Data 1

Supplementary Data 2

Supplementary Data 3

Supplementary Data 4

Supplementary Data 5

Supplementary Data 6

Supplementary Data 7

Supplementary Data 8

Supplementary Data 9

Supplementary Data 10

Supplementary Data 11

Supplementary Data 12

Supplementary Data 13

Supplementary Data 14

Supplementary Data 15

Supplementary Data 16

Reporting Summary

## Data Availability

The DNA sequence data used for phylogenetics are in an NCBI SRA repository under BioProject PRJNA819460; the rest of the data are provided as supplementary information.

## Code availability

Our codes are provided as supplementary information.
