# Supplementary material for: Geographic isolation drives speciation in Nearctic aphids
Source: Commun Biol. 2022 Aug 8;5:796. doi: 10.1038/s42003-022-03771-5 (PMC9360434; doi:10.1038/s42003-022-03771-5)
Supplement: Supplementary file 1 — Description of Additional Supplementary Files [file 42003_2022_3771_MOESM1_ESM.pdf]

Description of Additional Supplementary Files

File name: Supplementary Data 1
Description: Specimen data for samples used in the UCE-based phylogeny estimate.

File name: Supplementary Data 2
Description: Python script used to concatenate UCE alignments.

File name: Supplementary Data 3
Description: UCE-based phylogeny estimate, in Newick format.

File name: Supplementary Data 4
Description: COI barcode accession data.

File name: Supplementary Data 5
Description: : Phylogeny estimate combining UCE and COI data, in Newick format.

File name: Supplementary Data 6
Description: Aphid host-use data.

File name: Supplementary Data 7
Description: R script used to model niche features of aphid genera.

File name: Supplementary Data 8
Description: : R script used to model niches of aphid species.

File name: Supplementary Data 9
Description: New aphid specimen occurrence data provided by the Canadian National Insect Collection.

File name: Supplementary Data 10
Description: Combined aphid specimen occurrence data. (Combining data from the Canadian National Insect Collection with previously available data from GbiF and IdigBIO.)

File name: Supplementary Data 11
Description: Predictors used in path models of the species richness of aphid genera.

File name: Supplementary Data 12
Description: Predictors used in path models of aphid speciation rates.

File name: Supplementary Data 13
Description: Nearctic aphid phylogeny estimate with one tip per genus.

File name: Supplementary Data 14
Description: R code specifying and fitting path models of the species richness of aphid genera.

File name: Supplementary Data 15
Description: R code specifying and fitting path models of aphid speciation rates.

File name: Supplementary Data 16
Description: An overview of the analytical workflow.
