# Supplementary material for: Geographic isolation drives speciation in Nearctic aphids
Source: Commun Biol. 2022 Aug 8;5:796. doi: 10.1038/s42003-022-03771-5 (PMC9360434; doi:10.1038/s42003-022-03771-5)
Supplement: Supplementary file 18 — Reporting Summary [file 42003_2022_3771_MOESM18_ESM.pdf]

nature portfolio

Corresponding author(s): Nate B Hardy

Last updated by author(s): Jun 14, 2022

Reporting Summary

Nature Portfolio wishes to improve the reproducibility of the work that we publish. This form provides structure for consistency and transparency in reporting. For further information on Nature Portfolio policies, see our Editorial Policies and the Editorial Policy Checklist.

Statistics

For all statistical analyses, confirm that the following items are present in the figure legend, table legend, main text, or Methods section.

n/a	Confirmed	
☐	☒	The exact sample size (*n*) for each experimental group/condition, given as a discrete number and unit of measurement
☒	☐	A statement on whether measurements were taken from distinct samples or whether the same sample was measured repeatedly
☐	☒	The statistical test(s) used AND whether they are one- or two-sided<br>*Only common tests should be described solely by name; describe more complex techniques in the Methods section.*
☐	☒	A description of all covariates tested
☐	☒	A description of any assumptions or corrections, such as tests of normality and adjustment for multiple comparisons
☐	☒	A full description of the statistical parameters including central tendency (e.g. means) or other basic estimates (e.g. regression coefficient) AND variation (e.g. standard deviation) or associated estimates of uncertainty (e.g. confidence intervals)
☐	☒	For null hypothesis testing, the test statistic (e.g. *F*, *t*, *r*) with confidence intervals, effect sizes, degrees of freedom and *P* value noted<br>*Give P values as exact values whenever suitable.*
☒	☐	For Bayesian analysis, information on the choice of priors and Markov chain Monte Carlo settings
☒	☐	For hierarchical and complex designs, identification of the appropriate level for tests and full reporting of outcomes
☒	☐	Estimates of effect sizes (e.g. Cohen's *d*, Pearson's *r*), indicating how they were calculated

Our web collection on statistics for biologists contains articles on many of the points above.

Software and code

Policy information about availability of computer code

Data collection	No software was used to collect data.
Data analysis	DNA sequence processing was with the Snakemake pipeline (https://github.com/AAFC-BIOoE/snakemake-partial-genome-pipeline). Comparative statistical analyses were performed with R version 4.2.0, using the packages vegan 2.6-2, rgbif 3.7.2, raster 3.5-21, phylopath v1.1.3, phangorn v2.9.0, phytools 1.0-3, bestNormalize 1.8.3, and lavaan 0.6-12.

For manuscripts utilizing custom algorithms or software that are central to the research but not yet described in published literature, software must be made available to editors and reviewers. We strongly encourage code deposition in a community repository (e.g. GitHub). See the Nature Portfolio guidelines for submitting code & software for further information.

Data

Policy information about availability of data

All manuscripts must include a data availability statement. This statement should provide the following information, where applicable:

- Accession codes, unique identifiers, or web links for publicly available datasets
- A description of any restrictions on data availability
- For clinical datasets or third party data, please ensure that the statement adheres to our policy

The DNA sequence data used for phylogenetics are in an NCBI SRA repository under BioProject PRJNA819460; the rest of the data and codes are provided as supplementary information.

Human research participants

Policy information about studies involving human research participants and Sex and Gender in Research.

Reporting on sex and gender	NA
Population characteristics	NA
Recruitment	NA
Ethics oversight	NA

Note that full information on the approval of the study protocol must also be provided in the manuscript.

Field-specific reporting

Please select the one below that is the best fit for your research. If you are not sure, read the appropriate sections before making your selection.

☐ Life sciences ☐ Behavioural & social sciences ☒ Ecological, evolutionary & environmental sciences

For a reference copy of the document with all sections, see nature.com/documents/nr-reporting-summary-flat.pdf

Ecological, evolutionary & environmental sciences study design

All studies must disclose on these points even when the disclosure is negative.

Study description	This is a comparative phylogenetic analysis of the causes of variation in the species richness of aphid genera and the speciation rates of aphid lineaeges. We estimate phylogenetic relationships among more than 400 aphid species from target-enriched genomic data. We characterize aphid host-use niches from published ecological and specimen data, and we characterize aphid non-host-related niches by correlating aphid occurence data with several environmental variables. We then evaluate competing causal hypotheses with phylogenic path models.
Research sample	Our analyses are based on data from more than 400 Nearctic aphid species.
Sampling strategy	The sampling of aphid specimens was an opportunistic but concerted effort over more than a decade. We sought to sample as much of the Nearctic aphid fauna as was possible.
Data collection	Aphid specimens were collected in the field along with detailed information about provenance by experts in aphid systematics.
Timing and spatial scale	Sampling of aphid species took place over more than a decade, throughout much of Canada and the USA.
Data exclusions	No data were excluded from the analysis
Reproducibility	This is not experimental work. It is a comparative statistical analysis.
Randomization	NA
Blinding	NA

Did the study involve field work? ☐ Yes ☐ No

Field work, collection and transport

Field conditions	Variable.
Location	Much of Canada and the USA.
Access & import/export	Any sampling on public lands was in compliance with state and federal regulations.
Disturbance	Sampling of aphids is non-disruptive.

Reporting for specific materials, systems and methods

We require information from authors about some types of materials, experimental systems and methods used in many studies. Here, indicate whether each material, system or method listed is relevant to your study. If you are not sure if a list item applies to your research, read the appropriate section before selecting a response.

Materials & experimental systems

n/a	Involved in the study	
☒	☐	Antibodies
☒	☐	Eukaryotic cell lines
☒	☐	Palaeontology and archaeology
☐	☒	Animals and other organisms
☒	☐	Clinical data
☒	☐	Dual use research of concern

Methods

n/a	Involved in the study	
☒	☐	ChIP-seq
☒	☐	Flow cytometry
☒	☐	MRI-based neuroimaging

Animals and other research organisms

Policy information about studies involving animals; ARRIVE guidelines recommended for reporting animal research, and Sex and Gender in Research

Laboratory animals	NA
Wild animals	Our study is of the Nearctic fauna of aphids.
Reporting on sex	Most aphids are females, in some species males are entirely absent.
Field-collected samples	NA
Ethics oversight	NA

Note that full information on the approval of the study protocol must also be provided in the manuscript.
